# Transcriptome insights into newcastle disease virus-mediated eradication of cholangiocarcinoma cells

**DOI:** 10.1371/journal.pone.0322307

**Published:** 2025-05-06

**Authors:** Suphannee Thanyaphoo, Chanachai Sae-Lee, Wilasinee Thaopech, Warisa Amornrit, Mutita Junking, Pa-thai Yenchitsomanus, Naravat Poungvarin

**Affiliations:** 1 Clinical Molecular Pathology Laboratory, Department of Clinical Pathology, Faculty of Medicine Siriraj Hospital, Mahidol University, Bangkok, Thailand; 2 Research Division, Faculty of Medicine Siriraj Hospital, Mahidol University, Bangkok, Thailand; 3 Veterinary Biologics Assay and Research Center, National Institute of Animal Health, Department of Livestock Development, Ministry of Agriculture and Cooperatives, Nakhon Ratchasima, Thailand; 4 Siriraj Center of Research Excellence for Cancer Immunotherapy, Faculty of Medicine Siriraj Hospital, Mahidol University, Bangkok, Thailand; University of Veterinary and Animal Sciences, PAKISTAN

## Abstract

Newcastle Disease Virus (NDV) has emerged as a promising oncolytic viral therapy for various human cancers; however, its effectiveness against cholangiocarcinoma (CCA) remains unexplored. This study presents the capability of the lentogenic LaSota strain of NDV to eliminate two CCA cell lines, KKU-055 and KKU-100, as well as the potential molecular mechanisms underlying this effect. Comprehensive transcriptome analysis revealed alterations in gene expression within several pathways in CCA cells following exposure to the LaSota strain NDV, including those involved in TNF-alpha signaling via NF-kB, interferon alpha response, apoptosis, and IL-6/JAK/STAT3 signaling pathways. We remarkably observed a contrasting alteration in the expression of *CXCR4*, *GRAMD1B*, *IGFBP4*, and *TGM2* genes in KKU-055 and KKU-100 cells. In addition, gene network analysis highlighted *CCNA2*, *CDK1*, *DDX58*, *DHX58*, *EXO1*, *GBP1*, *IFIH1*, *IFIT1*, *IFIT2*, *IFIT3*, *IRF7*, *ISIG15*, *MX1*, *OAS1*, *OAS2*, *PARP9*, *TOP2A* and *XAF1* as potential hub genes influencing the response of CCA cells to NDV LaSota strain. Our findings offer evidence supporting the promise of NDV-based therapies as potential strategies for eliminating CCA cells.

## Introduction

In recent years, oncolytic viruses have gained attention as a potential cancer treatment due to their ability to target and eliminate cancer cells. Ideal oncolytic viruses possess several key characteristics. These viruses should not cause disease in humans or harm to healthy cells and tissues. These characteristics ensure that their use as therapeutic agents does not introduce additional health risks to the patients. These viruses should be able to specifically target cancer cells. Once inside the cancer cells, oncolytic viruses should have efficient ability to destroy cancer cells. In addition to directly targeting cancer cells, oncolytic viruses should effectively boost anti-tumor immune responses, resulting in further cancer cell eradication [1].

Newcastle disease virus (NDV) is an avian paramyxovirus. NDV is categorized into three pathotypes, which are determined by the severity of the disease it causes in avian species. These pathotypes are lentogenic (avirulent), mesogenic (intermediate), and velogenic (virulent) [2]. The lentogenic pathotype of NDV is characterized by its low pathogenicity, causing mild or asymptomatic infections in birds. The mesogenic pathotype exhibits moderate pathogenicity, leading to more noticeable disease symptoms and potential mortality. The velogenic pathotype, on the other hand, is highly virulent and can cause severe disease with high mortality rates in affected birds. Lentogenic NDV strains, recognized for their avirulence, are extensively used in avian vaccines and have a well-established history of safe application in veterinary medicine. NDV is safe for humans due to its host-specific pathogenesis. NDV infections in humans are rare and mild, as evidenced by observations in bird handlers and clinical studies utilizing NDV. A clinical study on NDV as an oncolytic agent in humans found that intravenous administration of 10^10^ PFU of lentogenic NDV-HUJ was safe and did not cause any adverse effects [3].

Studies have demonstrated that all pathotypes of NDV can cause oncolysis [4–7]. Several strains of NDV have shown the ability to specifically target cancer cells and induce lytic effects, resulting in the death of the cancer cells. Unlike other oncolytic viruses, NDV selectively enters, replicates, and mediates oncolysis in cancer cells through unique mechanisms [8–10]. The viral surface glycoproteins of natural NDV strains can bind to sialic acid-containing proteins, which are overexpressed on the surface of cancer cells, facilitating their entry. Additionally, cancer cells typically have impaired type I interferon signaling pathways, which are crucial for antiviral defense mechanisms, allows NDV to enter and replicate within cancer cells more effectively than in normal cells. In addition to causing direct cytotoxicity to host cells, NDV activates multiple signalling pathways, triggering cancer cell death by apoptosis, necrosis, and autophagy. Furthermore, NDV activates innate and adaptive anti-tumor immune responses, leading to the recruitment of immune cells that further attack and destroy cancer cells. NDV has been extensively investigated for its potential as an anticancer agent through *in vivo* studies [4,11–13]. NDV has been found to be well-tolerated and safe in clinical trials. Some patients responded to the treatment, demonstrating improved survival rates [1]. While NDV has shown promise in preclinical and clinical studies in certain types of cancer, its efficacy in eliminating CCA cells has not been reported.

The NDV LaSota strain is classified as a lentogenic strain and is commonly utilized in the production of Newcastle disease vaccines. It has also demonstrated notable effectiveness in eliminating cancer cells [5,14–16]. The NDV LaSota strain is cytotoxic to cancer cells at significantly lower doses than to normal human cells [17]. Its low pathogenicity makes it a safe and promising candidate for oncolytic virotherapy. In addition, avirulent lentogenic strains like LaSota are preferable for clinical use, as they avoid the risks associated with highly infectious strains, which could be unintentionally released into the environment.

Cholangiocarcinoma (CCA) is an epithelial cancer of the biliary duct system that may originate in the liver and extrahepatic bile ducts. High incidence and mortality rates have been reported in Eastern Asian and South-Eastern Asian countries, including Thailand [18,19]. CCA is often diagnosed at an advanced stage, making surgical removal challenging. Additional therapeutic approaches for CCA include stenting, radiation, chemotherapy and targeted therapy. Numerous ongoing research efforts are focused on improving existing CCA treatments, as well as developing new and more effective treatments with fewer side effects.

Poorly differentiated CCA is an aggressive malignancy with profound molecular complexity. This cancer type presents significant therapeutic challenges, with a critical unmet need for effective treatment strategies. In this study, we specifically assessed the effectiveness of the avirulent NDV LaSota strain, which has demonstrated therapeutic promise in various cancer types including colorectal, pancreatic, and breast cancers [17,20,21], in destroying poorly differentiated CCA cell lines. We also explored the underlying mechanisms for these effects, to evaluate the potential of NDV as a therapeutic agent for CCA.

## Materials and methods

### Production of NDV

Lentogenic NDV LaSota strain was inoculated in 9–11 days old specific pathogen-free embryonated chicken eggs, aseptically harvested from allantoic fluid, and then purified. Hemagglutination test using chicken red blood cells was performed to detect the presence of NDV and to determine NDV titer. NDV was aliquoted and stored at -80 °C until the start of the experiment.

### Cell culture

The KKU-100 (JCRB 1568) and KKU-055 (JCRB 1551) cell lines were purchased from the Japanese Collection of Research Bioresources Cell Bank. All cells were cultured and maintained in Dulbecco’s Modified Eagle’s Medium/Hams F-12 50/50 Mix (Gibco, USA), supplemented with 10% (v/v) fetal bovine serum (Hyclone laboratories, USA), under a humidified atmosphere of 5% CO_2_ at 37°C. These cells were tested for mycoplasma contamination by PCR-based assay before being used in experiments.

### Cell viability

Cells were seeded in six-well plates at a density of 3.5 x 10^5^ cells per well for KKU-055 and at a density of 4.0 x 10^5^ cells per well for KKU-100. Cells were cultured in complete media for 24 hours and infected with NDV with a dose of 16 HAU per 10,000 cells. Cells were trypsinized at 72 hours after infection, stained with trypan blue, and counted using the TC20 automated cell counter (Bio-Rad, USA). The number of cells was subsequently compared with that of the mock-treated cells.

### RNA extraction

Collected cells were preserved using RNAlater (Invitrogen, USA). RNA was isolated from these cells using the RNeasy Plus Mini Kit (Qiagen, Germany), according to the manufacturer’s instructions. The quality and quantity of RNA were assessed at Macrogen (Korea) using 2100 Bioanalyzer (Agilent Technologies, USA).

### RNA sequencing and analysis

Libraries were constructed by TruSeq stranded mRNA library preparation kit (Illumina, USA) and sequenced on an Illumina NovaSeq6000 at Macrogen (Korea). Processing and analysis of the raw read data with the trimming, quality control and alignment were performed on the Galaxy platform [22]. The Trimmomatic tool [23] was used to filter and trim low-quality reads, duplicated sequences, and adapter sequences. After filtering, the quality of the cleaned raw reads was assessed using FASTQC. The processed reads were aligned to the Homo sapiens genome (hg38) using HISAT2 with the default settings [24]. Transcriptome qualification was performed using featureCounts to calculate transcript read counts [25].

Differential expression analysis was conducted using limma-Voom [26,27]. The MSigDB hallmark [28] enrichment analysis was performed on differentially expressed genes using a threshold of an absolute log2 fold change > 2 and an adjusted p-value < 0.05. This analysis was executed using enrichR [29–31], R and the GSEA dataset. We use STRING for protein–protein association networks and functional enrichment analysis [32]. The Cytoscape and CytoHubba plugin were used to identify the hub genes on the protein–protein interaction network [33].

### Statistical analysis

The mean between two groups was compared using the Student’s t test. Data are presented as mean±SD, and p-value or adjusted p-value < 0.05 was considered significant.

## Results

### The LaSota strain of NDV significantly reduced the number of two CCA cells

**Fig 1:**
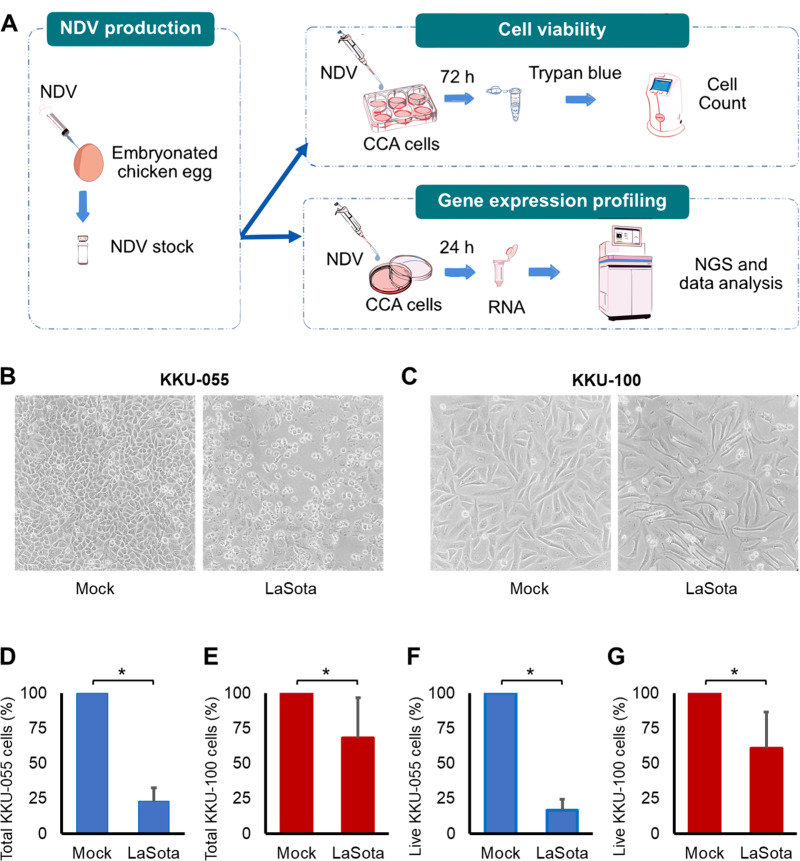
Effect of NDV LaSota strain in KKU-055 and KKU-100 CCA cells. (A) Overview of the experiments conducted to assess the impact of NDV in KKU-055 and KKU-100 CCA cell lines. (B-C) Representative bright-field microscopy images of CCA cells. Cells were cultured for 24 hours in complete media before being infected with a viral dilution of 16 HAU per 10,000 cells. Images depicting KKU-055 (B) and KKU-100 (C) CCA cells at 72 hours after NDV infection.(D-G) Evaluation of cell number after NDV infection. Cells were trypsinized, stained with trypan blue, and counted at 72 hours after infection. Bar graphs show the percentage of total KKU-055 cells (D), total KKU-100 cells (E), live KKU-055 cells (F) and live KKU-100 cells (G) in comparison to the mock controls (* p < 0.05). The data presented were acquired from three experiments, each comprising three replicates within each condition.

Our study examines the effect of the NDV LaSota strain on two distinct human CCA cell lines to reveal potential variations in the impact of this NDV in different CCA contexts. Specifically, we utilized KKU-055 and KKU-100 cell lines, both derived from Opisthorchis viverrini-related poorly differentiated CCA cells. KKU-055 originated from intrahepatic CCA in a 56-year-old Thai male, while KKU-100 was derived from perihilar CCA in a 65-year-old Thai female [34]. It has been demonstrated that KKU-100 is less sensitive to chemotherapeutic drugs compared to KKU-055 [35]. An overview of our experimental design is illustrated in Fig 1A.

We first assessed the oncolytic capabilities exhibited by the NDV LaSota strain in both CCA cells. At 72 hours following NDV LaSota strain infection, the KKU-055 cell population exhibited a decrease to 23.31% (Fig 1B and 1D), while the KKU-100 cell population showed a comparatively moderated decrease to 68.46%, relative to the mock-treated control group. (Fig 1C and 1E). Trypan blue staining was used to exclude permeable dead cells from our analysis. We found that the number of live cells, remaining unstained with trypan blue, was 16.77% for KKU-055 cells and 60.75% for KKU-100 cells, both compared to the mock-treated control (Fig 1F and 1G).

Our data clearly demonstrates the potential of the NDV LaSota strain to effectively eliminate CCA cells. Two CCA cell lines used in our study exhibit varying levels of cell survival following NDV treatment. This notable difference emphasizes the requirement for in-depth investigations into the complex molecular alterations occurring within CCA cells due to the influence of the NDV LaSota strain.

### Transcriptome sequencing analysis revealed gene expression patterns and pathways in the NDV-infected CCA cells

**Fig 2:**
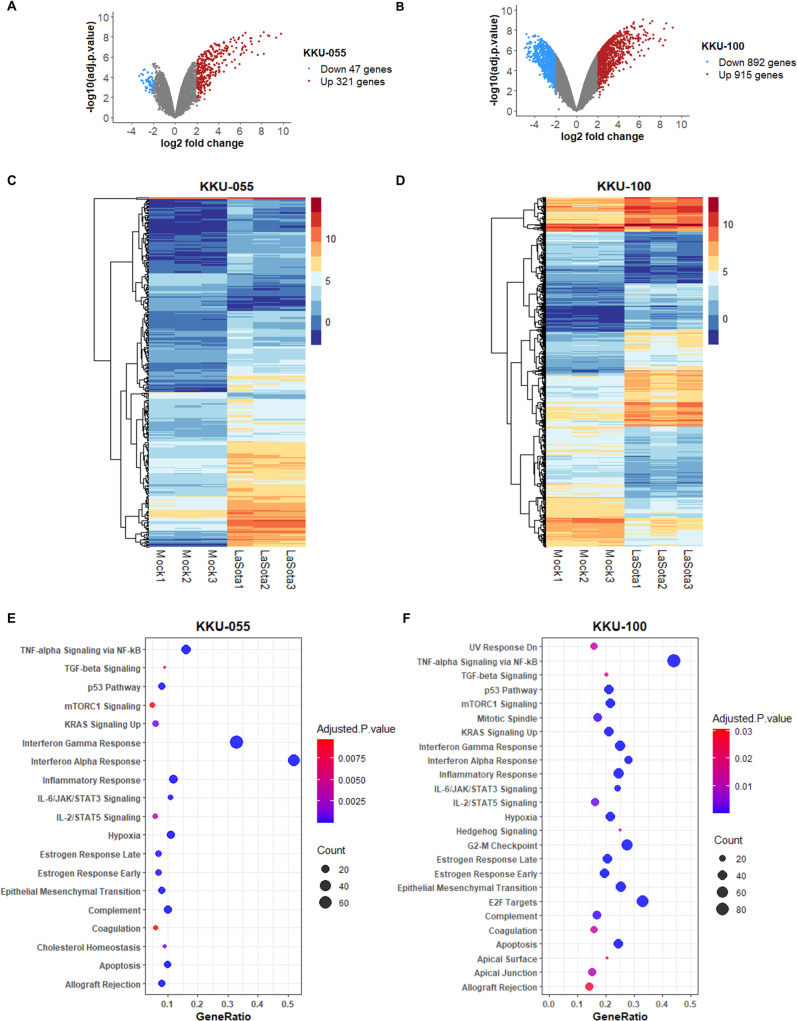
Differential expression and pathway analysis of RNA-seq data in NDV LaSota strain-treated CCA cells. (A-B) Volcano plots, generated by the Limma-voom tool in the Galaxy-based platform, illustrate the differential expression patterns in KKU-055 (A) and KKU-100 (B) CCA cells at 24 hours after NDV LaSota strain infection. Significantly upregulated and downregulated genes in comparison to mock controls (adjusted p-value < 0.05) are represented by red and blue dots, respectively. (C-D) Heat maps of the differentially expressed genes in KKU-055 (C) and KKU-100 (D) cells after NDV LaSota infection. The range of expression levels is represented by the colors. (E-F) Pathways significantly enriched by hallmark analysis in KKU-055 (E) and KKU-100 (F) cells following NDV LaSota strain exposure.

RNA sequencing was utilized to investigate alteration of gene expression profiles after NDV LaSota strain infection in both KKU-055 and KKU-100 cells. Compared to the mock control group, the treatment of KKU-055 cells with the NDV LaSota strain resulted in a significant differential expression of 368 genes (Fig 2A and 2C; S1 Table). Among these genes, 321 genes were found to be upregulated while 47 genes were downregulated in the NDV-treated KKU-055 cells. In the KKU-100 cells, a total of 1,807 genes exhibited significant differential expression when compared to the control group (Fig 2B and 2D; S2 Table). Out of these genes, 892 genes were upregulated, while 915 genes were downregulated in the KKU-100 cells following the NDV treatment.

To gain biological insights into the pathways associated with differentially expressed genes, we conducted a hallmark pathway enrichment analysis. The analysis of 368 altered genes in KKU-055 cells after NDV LaSota strain infection uncovered 19 enriched pathways. (Fig 2E; S3 Table). In KKU-100 CCA cells post NDV LaSota strain infection, a total of 1,807 genes were associated with 25 enriched pathways (Fig 2F; S3 Table). Notably, the cholesterol homeostasis pathway was found to be significant only in KKU-055 cells, whereas KKU-100 cells exclusively displayed G2-M checkpoint, apical surface, E2F targets, UV response Dn, mitotic spindle, hedgehog signaling, and apical junction pathways.

Pathways that are significantly enriched in both CCA cells are IL-2/STAT5 signaling, mTORC1 signaling, p53 pathway, interferon gamma response, allograft rejection, epithelial mesenchymal transition, estrogen response early, inflammatory response, apoptosis, TNF-alpha signaling via NF-kB, estrogen response late, KRAS signaling Up, TGF-beta signaling, complement, coagulation, IL-6/JAK/STAT3 signaling, hypoxia, and interferon alpha response.

These findings highlight the potential involvement of multiple pathways in the disparate responses of the two CCA cells to the NDV LaSota strain. Additional analysis of RNA-seq results to pinpoint important genes within gene expression networks could enhance our understanding of the underlying molecular mechanisms that transpire after NDV exposure in these CCA cells.

### Oncolytic potential of NDV in CCA cells is linked to several pivotal genes

**Fig 3:**
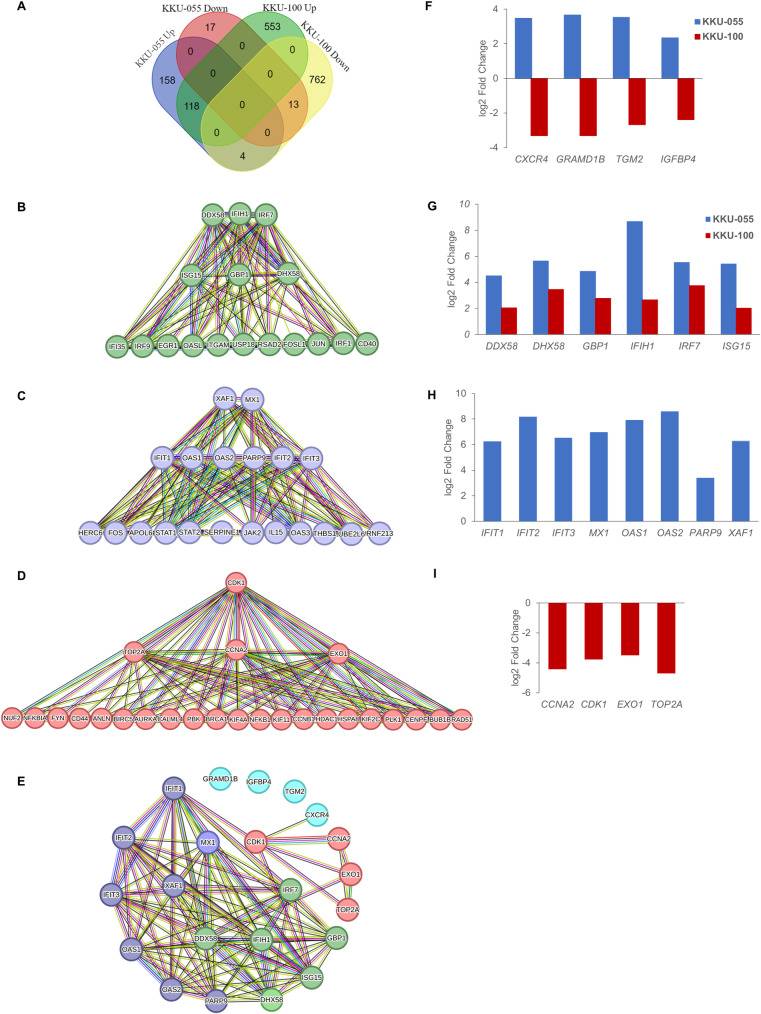
Potential key genes associated with oncolytic impact of NDV LaSota strain in CCA cells. (A) Venn diagram illustrates the number of genes altered in KKU-055 and KKU-100 cells following 24 hours of exposure to NDV LaSota strain. The analysis solely incorporates genes identified in the RNA sequencing results of both cells. (B-D) Hub genes identified based on protein-protein interaction network. The top 10 genes identified and ranked using five methods including node degree, maximal clique centrality (MCC), maximum neighborhood component (MNC), betweenness, and closeness, were selected. The hub genes identified by node degree, MCC, and MNC are shown in the topmost and middle rows. The topmost row displays hub genes identified by all five methods. Hub genes were identified from 3 groups of genes: genes with the same expression pattern in KKU-055 and KKU-100 cells (B), genes exclusively altered in KKU-055 cells (C), and genes exclusively altered in KKU-100 cells (D). (E) Association among 6 hub genes with the same expression pattern in KKU-055 and KKU-100 cells, 8 hub genes exclusively altered in KKU-055 cells, 4 hub genes exclusively altered in KKU-100 cells and 4 genes altered in opposite directions in two CCA cells. (F-I) Gene expression changes compared between NDV LaSota- and mock- treated cells. The bar graph illustrates differential expression of the genes exhibited significant changes in expression: 4 genes altered in opposite directions in two CCA cells (F), 6 hub genes with the same expression pattern in KKU-055 and KKU-100 cells (G), 8 hub genes exclusively altered in KKU-055 cells (H), and 4 hub genes exclusively altered in KKU-100 cells (I).

We conducted a comparison of gene expression profiles following exposure to the NDV LaSota strain in KKU-055 and KKU-100 CCA cells. The genes included in this comparative assessment were exclusively those detected in the RNA sequencing results of both cells. KKU-055 cells exhibited 280 upregulated genes and 30 downregulated genes following infection with the NDV LaSota strain, while KKU-100 cells demonstrated 671 upregulated genes and 779 downregulated genes (Fig 3A). Interestingly, we found a set of four genes that exhibited upregulation in KKU-055 cells and downregulation in KKU-100 cells. These genes are C-X-C motif chemokine receptor 4 (*CXCR4*), GRAM domain containing 1B (*GRAMD1B*), insulin like growth factor binding protein 4 (*IGFBP4*) and transglutaminase 2 (*TGM2*) genes (Fig 3F).

Following NDV LaSota strain infection, 131 genes were altered in both KKU-055 and KKU-100 cells. These shared gene changes represent a common molecular shift within both cells. Comprehensive bioinformatics analysis reveals a total of 6 hub genes. They are DExD/H-box helicase 58 (*DDX58*), interferon induced with helicase C domain 1 (*IFIH1*), interferon regulatory factor 7 (IRF7), ISG15 ubiquitin like modifier (*ISG15*), guanylate binding protein 1 (*GBP1*), and DExH-box helicase 58 (*DHX58*) (Fig 3B and 3G; S4 Table). Notably, the expression of these genes was increased in both CCA cells, and the log2 fold changes for these genes in KKU-100 cells were lower in comparison to those in KKU-055 cells.

Another set of 8 hub genes has been identified through an analysis within a pool of 175 genes exclusively altered in KKU-055 cells following exposure to NDV LaSota strain. These genes are XIAP associated factor 1 (*XAF1*), MX dynamin like GTPase 1 (*MX1*), interferon induced protein with tetratricopeptide repeats 1 (*IFIT1*), interferon induced protein with tetratricopeptide repeats 2 (*IFIT2*), interferon induced protein with tetratricopeptide repeats 3 (*IFIT3*), 2’-5’-oligoadenylate synthetase 1 (*OAS1*), 2’-5’-oligoadenylate synthetase 2 (*OAS2*), and poly (ADP-ribose) polymerase family member 9 (*PARP9*) (Fig 3C and 3H; S4 Table). The expression of each of these genes was upregulated. Among the 1,315 genes exclusively altered within NDV LaSota-infected KKU-100 cells, four pivotal hub genes have been identified. These genes include cyclin dependent kinase 1 (*CDK1*), DNA topoisomerase II alpha (*TOP2A*), cyclin A2 (*CCNA2*), and exonuclease 1 (*EXO1*) (Fig 3D and 3I; S4 Table). All of them exhibited downregulated expression.

The interaction network among all predicted hub genes is illustrated in Fig 3E. Identification of hub genes highlights the potential involvement of multiple critical genes in NDV-mediated oncolysis within CCA cells, contributing to a better understanding of NDV’s therapeutic prospects in the context of CCA treatment.

## Discussion

The investigation of oncolytic therapy for cholangiocarcinoma is still in the early stages. Several studies are underway to enhance the utilization of NDV strains and investigate the mechanisms underlying their anti-cancer properties. The lentogenic LaSota strain of NDV has been shown to have promising oncolytic activity against various cells including pancreatic, breast, and cervical cancer cells [5,14–16]. To the best of our knowledge, no previous studies have explored the efficacy of any NDV strain as an oncolytic agent in CCA. This study presents the initial evidence demonstrating the oncolytic potential of NDV LaSota strain in two CCA cell lines.

NDV-mediated oncolysis is more effective in KKU-055 cells than in KKU-100 cells. This finding aligns with a prior investigation that noted distinct sensitivities to NDV among diverse cell lines [4]. This observation implies that the response to NDV treatment is potentially influenced by the unique cell characteristics or alterations. Our transcriptome sequencing analysis identified distinct differences in the gene expression profiles of two CCA cell lines that exhibited varying sensitivity to the LaSota strain of NDV. *CXCR4, GRAMD1B*, *IGFBP4,* and *TGM2* were genes which displayed contrasting upregulation in KKU-055 cells and downregulation in the KKU-100 cells. Previous study demonstrated that *GRAMD1B* significantly inhibits cell migration and promotes cell death by deactivating JAK/STAT signaling in breast cancer [36]. The *IGFBP4* gene is a member of the insulin-like growth factor system that inhibits various cancer cells *in vitro* and *in vivo*. Overexpression of *IGFBP4* diminished the growth of prostate cancer [37], and colorectal cancers [38]. The *TGM2* gene, also known as TG2, can induce apoptosis in pancreatic ductal adenocarcinoma cells [39]. These findings are in line with our results that the *GRAMD1B*, *IGFBP4*, and *TGM2* genes are upregulated in KKU-055 cells, which exhibit lower cell survival rates following NDV infection. High expression of CXCR4 was reported in KKU-100 cells and stimulation of CXCR4 promoted cancer cell migration and invasion [40]. Silencing of *CXCR4* in the hilar CCA cell line QBC939 led to decreased cell proliferation [41]. *CXCR4* expression was found to be correlated with intrahepatic CCA progression and metastasis. Abrogation of *CXCR4* significantly reduced intrahepatic CCA cell proliferation, cell cycle, cell invasion, and tumorigenicity [42]. Further investigations are required to explore the effects of *CXCR4* upregulation in KKU-055 cells and *CXCR4* downregulation in KKU-100 cells on their survival rates following exposure to NDV LaSota strain.

NDV is known to activate several signaling pathways, including apoptosis (intrinsic, extrinsic and execution pathways), necrosis/necroptosis, autophagy, and stress signaling (endoplasmic reticulum stress and stress-activated mitogen-activated protein kinase pathways) [43]. Our pathway enrichment analysis revealed the potential involvement of several pathways in CCA cells in response to NDV LaSota strain treatment. It is worth noting that although these two CCA cell lines share certain pathways in their response to NDV LaSota strain infection, the specific genes within those pathways that are affected differ between the two cells. Enriched pathways, including TNF-alpha signaling via NF-kB, interferon alpha response, apoptosis, and IL-6/JAK/STAT3 signaling pathways, are consistent with results reported in previous studies [44–47].

The ability of the virus to lyse cancer cells, as well as the susceptibility of cancer cells to viral infection, are two factors that contribute to the oncolytic impact of NDV. In our RNA sequencing results, expression of *ST6GAL1* was not detected in both KKU-055 and KKU-100 cells. Wilden *et al.* identified an inverse relationship between the expression of antiviral genes, RIG-I (or *DDX58*), IRF-3, IRF-7, and IFN-β, and the susceptibility of cells to NDV infection [48]. Interestingly, we found that the expression of RIG-I and IRF-7 in KKU-100 cells is significantly higher than in KKU-055 cells. Additional investigation is needed to determine whether the expression levels of these genes influence the susceptibility of KKU-055 and KKU-100 cells to NDV infection.

Our *in silico* predictions identify hub genes that may be important in CCA cell responses to NDV LaSota strain exposure. All the hub genes identified from a subset of genes exclusively altered in KKU-055 cells and another subset altered in both CCA cell types are upregulated and recognized for their involvement in antiviral responses, including *DDX58*, *DHX58*, *GBP1*, *IFIH1*, *IFIT1*, *IFIT2*, *IFIT3*, *IRF7*, *ISIG15*, *MX1*, *OAS1*, *OAS2*, *PARP9*, and *XAF1*. Hub genes that exhibit downregulation in KKU-100 cells are *CCNA2*, *CDK1*, *EXO1*, and *TOP2A*. Silencing of these genes leads to reduced cancer cell proliferation. *CCNA2* knockdown inhibits colon cancer cell proliferation by impairing cell cycle progression and promotes apoptosis [49]. *CDK1* knockdown inhibits CCA development through cell proliferation, colony formation, cell migration and cell apoptosis [50], as well as mitigates the impact of *NUCKS1* overexpression on cell proliferation, invasion, and migration in non-small cell lung cancer [51]. Silencing of *EXO1* disrupts the cell cycle in hepatocellular carcinoma cells and reduces cell proliferation [52]. *TOP2A* knockdown inhibits cell proliferation and migration in pancreatic cancer cell lines [53].

There are a few limitations in our study. Firstly, we only investigated the oncolytic potential of one strain of NDV, which might not fully represent the diversity of NDV strains available. Additional studies using a wider range of NDV strains would provide a more comprehensive understanding of their effectiveness in treating cholangiocarcinoma. Secondly, we administered a single dosage of NDV in our experiments. Different dosages of NDV might yield varying results in terms of their oncolytic activity. It is important to explore different dosages to determine the optimal therapeutic concentration that can effectively target and destroy CCA cells while minimizing potential side effects. Lastly, our study focused on the analysis of two CCA cell lines. CCA is a heterogeneous disease with diverse molecular characteristics. Limiting our investigation to only two cell lines might not fully capture the overall response of various CCA subtypes to NDV treatment. Including a larger panel of CCA cell lines, representing different molecular subtypes, would provide a more comprehensive understanding of the effectiveness of NDV in different contexts.

## Conclusions

In summary, our study is the first to investigate the potential therapeutic value of NDV in CCA. We found the LaSota strain was effective in destroying KKU-055 and KKU-100 CCA cells. Our study presents alterations of several genes and pathways potentially associated with NDV’s oncolytic activities. Additional investigation is essential to establish the pivotal involvement of these genes and pathways in influencing the diverse response patterns of the CCA cell lines to NDV LaSota strain treatment.

## Supporting information

S1 Table List of differential expression genes in KKU-055 CCA cells after treatment with NDV LaSota strain. (XLSX)

S2 Table Genes that exhibited significant differential expression in KKU-100 CCA cells following treatment with NDV LaSota strain, compared to mock treatment control (log2 fold change > 2 and adjusted p-value < 0.05). (XLSX)

S3 Table Hallmark pathway enrichment analysis in KKU-055 and KKU-100 CCA cells following treatment with NDV LaSota strain (adjusted p-value < 0.05). (XLSX)

S4 Table Hub gene prediction in KKU-055 and KKU-100 CCA cells following treatment with NDV LaSota strain. (XLSX)
